# Genetic approaches for assessment of phosphorus use efficiency in groundnut (*Arachis hypogaea* L.)

**DOI:** 10.1038/s41598-022-24016-9

**Published:** 2022-12-13

**Authors:** Sai Rekha Kadirimangalam, Yashoda Jadhav, K. V. Nagamadhuri, Latha Putta, Tharanya Murugesan, Murali T. Variath, Anil Kumar Vemula, Surendra Singh Manohar, Sunil Chaudhari, Sunita Choudhary, Jana Kholova, Janila Pasupuleti

**Affiliations:** 1 International Crops Research Institute for the Semi-Arid Tropics (ICRISAT), Hyderabad, Telangana 502324 India; 2 Institute of Frontier Technology, Regional Agricultural Research Station, Acharya N.G. Ranga Agricultural University, Tirupati, Andhra Pradesh 517502 India

**Keywords:** Physiology, Plant sciences

## Abstract

Production of phosphorus efficient genotypes can reduce environmental pollution. Identification of *P*-efficient groundnut genotypes is a need of the hour to sustain in *P*-deficient soils. The pot experiment showed significant differences between genotypes (G) and treatments (T) for all the traits and G × T interaction for majority of traits. The G × T × Y interaction effects were also significant for all the traits except leaf P% (LP%), leaf acid phosphatase (LAP) and root dry weight (RDW). In lysimeter experiment, the effect of G, T and G × T were significant for leaf dry weight (LDW), stem dry weight (SDW), total transpiration (TT) and transpiration efficiency (TE). For traits, LDW, SDW, TT, TE, ICGV 00351 and ICGS 76; for SDW, TT, ICGV 02266 are best performers under both *P*-sufficient and deficient conditions. Based on P-efficiency indices and surrogate traits of *P*-uptake, ICGV’s 02266, 05155, 00308, 06040 and 06146 were considered as efficient *P*-responding genotypes. From GGE biplot, ICGV 06146 under *P*-deficient and TAG 24 under both *P*-sufficient and deficient conditions are portrayed as best performer. ICGV 06146 was identified as stable pod yielder and a promising genotype for *P*-deficient soils. The genotypes identified in this study can be used as a parent in developing mapping population to decipher the genetics and to devleop groundnut breeding lines suitable to P-deficient soils.

## Introduction

Mineral nutrients required for the plant growth are acquired from the soil and the macronutrients such as, Nitrogen (N), Phosphorus (P), Potassium (K), Calcium (Ca), Magnesium (Mg) and Sulphur (S) are the key components of organic compounds of the plants. Soil fertility is one of the important aspects of crop productivity and excess of nutrients leads to toxicity and lack of nutrients leads to a deficiency which poses a severe impact on crop growth^1^. Soil nutrient deficiency remain as a key constraint to crop production across the cropping systems and thus application of suitable amounts of nutrients to the soil at the correct time to improve crop yield has been a widely adopted practice. Integrated nutrient management practices ensure restoration and sustenance of soil fertility, have favorable effect on physical, chemical, and biological properties of soil and economizes fertilizer use. Cultivation of nutrient use efficient crop cultivars is a key component of integrated nutrient management as efficient cultivars can uptake and translocate the mineral nutrients from deficient soils to realize the potential crop yields and offer environmentally sustainable solutions.

Phosphorus is the second most important limiting macronutrient in the soil for plant growth after Nitrogen^2^. *P* is a key component of cell molecules such as nucleic acids, phospholipids, and adenosine triphosphate (ATP) and vital for all plant processes including root development, nitrogen fixation, photosynthesis and crop maturation^3^. *P* in the soil is present in both, organic and inorganic forms, of which 20–80% is available in organic form mainly as phytic acid^4^, while the remaining 20% is present as inorganic *P* in 170 different mineral forms^5^. *P* is taken up from the soil solution by plant roots mainly as primary orthophosphate ions (H_2_PO_4_^-^) and to a lesser extent as secondary orthophosphate ion (HPO_4_^2-^). Water stress limits the P uptake by the plants and will have consequences on plant growth^6^.

Globally, more than 40% of arable land is deficient in Phosphorus, and most of it falls in tropical and subtropical regions^7^. External application of *P* fertilizer as rock phosphate (water-insoluble) and triple superphosphate (water-soluble) is recommended in P-deficient soils. However, due to the low recovery of applied *P*, the plants remain largely unaffected to the applied *P*. Around 80% of the applied *P* fertilizer is lost due to precipitation, adsorption, or conversion of inorganic to organic form of *P* that is unavailable to plants^5^. The applied *P* is bound to the soil building a pool of *P* residues, or it may lose due to erosion, runoff or leaching and causes eutrophication and, in contrast, if not leached it may be retained by sorption or precipitation as (Fe and Al) hydroxides and (Ca) carbonates^8^.

Climate change is also likely to tailor the *P* availability from land to water and their ecological influence and these effects are indeterminate^9^. The changing climate would likely aggravate the *P* deficit soils. Main climatic effects such as high precipitation and high temperature enable rapid immobilization, mineralization, and weathering through direct effect or indirect effects on key soil properties and microbial activities, instigating changes to soil P forms and availability^10–13^. The increasing soil temperatures due to climate change leads to increasing *P* mineralization which affects the nutrient use efficiency by impacts on the influx rate of nutrient ions^14^. On the other hand, Phosphorus fertilizer demand is projected to increase due to the increasing population^15^. Most phosphorus fertilizers use rock phosphate as the main ingredient for manufacturing, and if the use continues at same level the current global levels of rock phosphate may be depleted in 50–100 years.

The groundnut or peanut (*Arachis hypogaea* L.) is an important grain legume crop grown in semi-arid tropics where *P*-deficiency is widespread. In Africa, among the soil nutrients, *P* is considered as a key constraint to the crop production^16^. Groundnut is an important source of protein, fat and micronutrients to humans. The kernels are rich in fat (~ 50%), protein (~ 25%), minerals, vitamins and antioxidants making them a valuable source for human nutrition. It is grown in an area of 31.56 million hectares with a total production of 53.63 million tons of pods^17^. Africa and Asia constitute > 90% of groundnut area where the production is challenged by abiotic stresses combined with biotic stresses, and poor soil fertility. Majority of the groundnut growing regions in the tropics and semi-arid tropics have soils with low *P* availability and are reported to face significant yield losses due to *P* deficiency^18^^,^^19^. In groundnut, *P* is essential for shoot growth, root growth, pod filling, enhancing the maturity of crop and fixation of atmospheric nitrogen through nodules. Because of this importance of *P*, identification of genetic variability for adaptation to *P*-deficient conditions is required to sustain *P* resources^20^.

Studies have shown an array of adaptive strategies to cope with limited *P* availability and allow efficient *P* acquisition in different crop species^21^. Under low *P* supply, the root to shoot ratio increased in maize and wheat significantly^22^^,^^23^. Whereas in rapeseed, *P*-deficiency triggered root length and root hair density^24^. Leaf acid phosphatase enhanced under *P*-deficient condition has been reported in various crop plants including rice, wheat, barley, clover and lupine^25^^,^^26^^,^^27^^,^^28^^,^^29^ and suggested that the leaf acid phosphatase could be used as diagnostic criterion for *P*-deficiency^30^^,^^26^. In groundnut the response to *P* not only depends on surrogate traits such as leaf acid phosphatase^30^^,^^31^, root length^32^, root hairs and gynophores^33^ but also depends on soil (soil type, the form of *P* availability and *P* solubilizing microorganisms in the soil^34^ and management practices. In this study, we attempt to identify P-related traits that can be exploitable in groundnut breeding programs to select *P*-efficient lines, and to identify the genotypes that show stable yield performance under *P*-sufficient and *P*-deficient conditions to be utilized in groundnut improvement programs. The main objective of this study was to screening of advanced breeding lines of groundnut and ascertaining the factors leading to difference between *P*-efficient and inefficient genotypes. This study also aims to quantify *P* use efficiency in groundnut cultivars and group them in terms of their efficiency and *P* availability. This study will identify genotypes to support the future breeding of groundnut cultivars for low *P* soil conditions, reducing inputs and improves the sustainability of production.

## Results

### ANOVA from pot experiment

Year-wise individual ANOVA showed significant differences for all the traits among the genotypes in 2016 and 2017, (Table 1). The treatment effect (i.e., *P*-sufficient and *P*-deficient) was also significant for all the traits in both the years except for RL and R:S ratio during rainy 2016. Genotype × treatment interaction effects were also significant for all the traits in both the years except for AC and RDW during rainy 2017.

**Table 1:** Year-wise analysis of variance (F- value) for phosphorus use efficiency and yield related traits in groundnut.

Source of variation	df	LP%	LAP	AC	RL	SL	R: S	RDW	PYP
**2016**									
Treatment	1	64.04***	68.66***	60.95***	0.04	17.01***	2.24	11.59***	108.15***
Rep (Treatment)	2	1.25	10.02***	0.60	0.59	0.48	1.44	1.14	2.30
Genotype	19	3.16**	5.27***	5.39***	77.68***	22.75***	33.60***	49.83***	23.60***
Genotype × Treatment	19	3.63***	2.18*	3.75***	44.36***	19.77***	24.75***	17.37***	15.70***
**2017**									
Treatment	1	49.53***	70.03***	19.25***	58.42***	45.65***	147.12***	47.29***	68.61***
Rep (Treatment)	2	3.45*	18.20***	3.66*	0.29	0.37	0.34	12.67***	2.21
Genotype	19	2.78**	4.49***	2.29*	33.67***	4.76***	17.55***	10.71***	8.80***
Genotype × Treatment	19	2.02*	2.00*	0.34	9.51***	1.94*	3.57***	1.17	7.87***

*: significant at < 0.05, **: significant at < 0.01, ***: significant at < 0.001 probability level.

df—Degrees of freedom, LP% –Leaf phosphorus (%), LAP—Leaf acid phosphatase (µM/hr/gm), AC—Anthocyanin content (mg/g), RL—Root length (cm), SL –S length (cm), R: S—Root: Shoot ratio, RDW—Root dry weight (g), PYP—Pod yield per plant (g).

Combined ANOVA revealed significant differences among the genotypes for all the traits under study (Table 2). The treatment effect was significant for all the traits except SL and the year effect was significant for all the traits except LAP. The genotype × treatment (G × T) interaction effects are significant for all the traits except AC whereas, the year × treatment (Y × T) interaction effect was significant for RL, SL, R:S ratio and RDW and genotype × year (G × Y) interactions are significant for all the traits except LP% and LAP. The genotype × treatment × year (G × T × Y) interaction effects are significant for AC, RL, SL, R:S ratio and PYP.

**Table 2:** Pooled ANOVA (F value) for phosphorus related and yield traits in groundnut.

Source of variation	df	Mean sum of squares							
		LP%	LAP	AC	RL	SL	R: S	RDW	PYP
Year	1	11.01***	3.88	4.18*	644.58***	393.87***	1039.35***	161.46***	19.99***
Treatment	1	113.39***	138.67***	72.05***	33.40***	1.48	88.59***	56.90***	171.03***
Replication (Year*Treatment)	4	2.31	13.86***	2.09	0.43	0.42	0.88	6.77***	2.22
Genotype	19	4.83***	9.40***	3.05***	62.74***	22.03***	23.76***	21.89***	25.39***
Year × Treatment	1	0.90	0.00	3.90	36.41***	56.44***	121.57***	32.41***	0.35
Year × Genotype	19	1.01	0.38	4.34***	40.05***	8.45***	20.65***	6.83***	5.19***
Genotype × Treatment	19	5.03***	3.98***	1.70	27.82***	13.06***	9.63***	3.68***	20.48***
Genotype × Treatment × Year	19	0.92	0.21	2.08*	19.28***	11.60***	9.80***	1.63	2.12*

*: significant at < 0.05, **: significant at < 0.01, ***: significant at < 0.001 probability level.

df—Degrees of freedom, LP%—Leaf phosphorus (%), LAP—Leaf acid phosphatase (µM/hr/gm), AC—Anthocyanin content (mg/g), RL—Root length (cm), SL –Shoot length (cm), R: S—Root: Shoot ratio, RDW—Root dry weight (g), PYP—Pod yield per plant (g).

### ANOVA from lysimeter experiment

Table 3 showed significant genotypic effect, treatment effect and genotype x treatment interaction effect for all the physiological traits studied under lysimeter experiment.

**Table 3:** ANOVA for physiological and yield related traits in groundnut under lysimeter.

Source of variation	df	LDW	SDW	TT	TE
		Mean sum of squares			
Genotype	19	9.01***	11.20***	6.65***	4.54***
Treatment	1	5.55*	30.49***	9.02*	18.10***
Genotype × Treatment	19	1.75*	2.20**	1.79*	1.92*

*: significant at < 0.05, **: significant at < 0.01, ***: significant at < 0.001 probability level.

LDW- Leaf dry weight (g); SDW—Stem dry weight (g); TT—Total transpiration per plant (g); TE—Transpiration efficiency (g/kg).

### Performance of groundnut genotypes for physiological traits from lysimeter experiment

**Figure 1:**
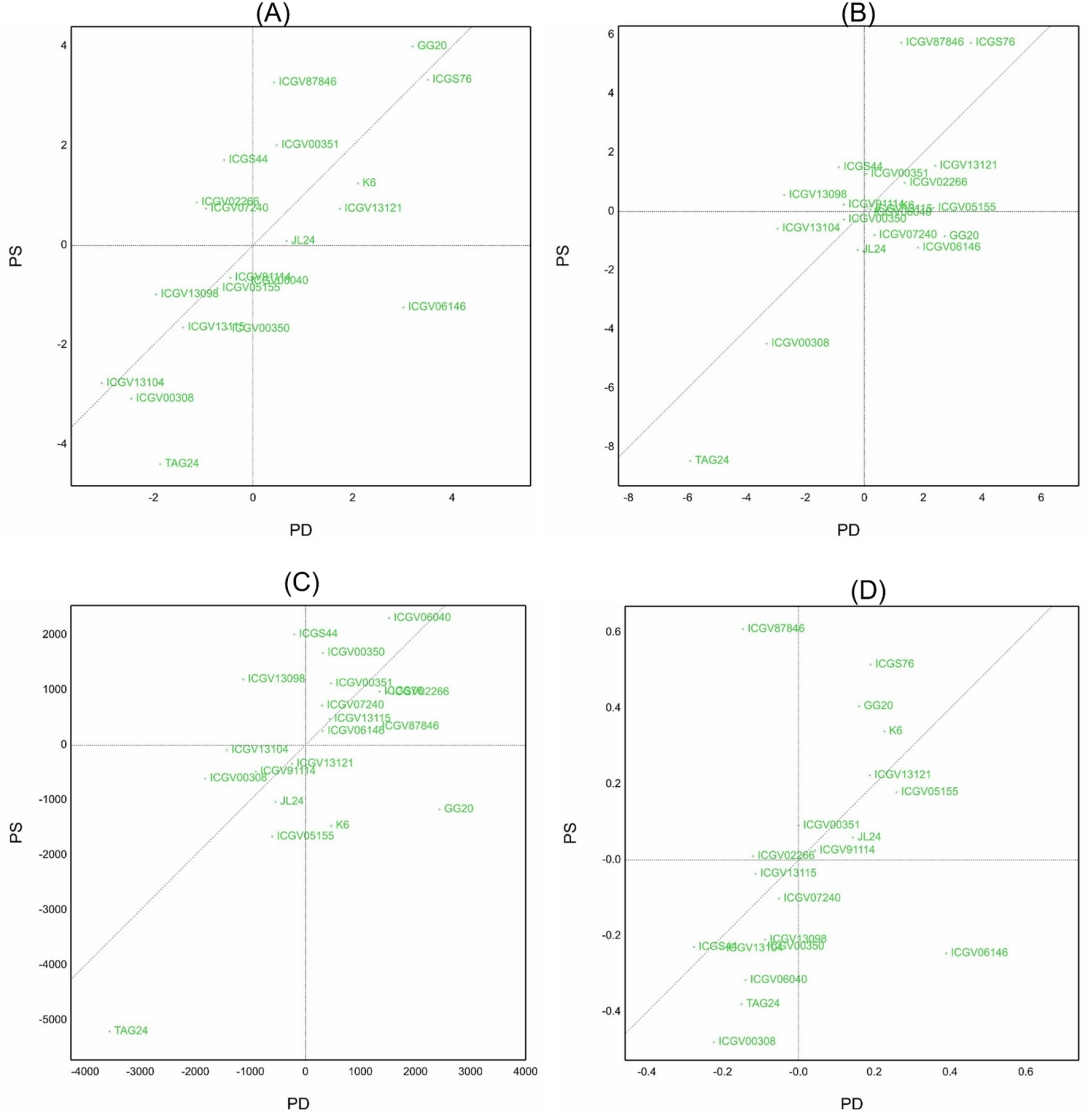
(**a**) Scatter plot showing distribution of genotypes for various traits under lysimeter experiment (**A**) Leaf dry weight (g) (**B**) stem dry weight (g). PD—Phosphorus deficiency; PS—Phosphorus sufficiency. (**b**) Scatter plot showing distribution of genotypes for various traits under lysimeter experiment (**C**) Total transpiration (g/kg) (**D**) Transpiration efficiency (g). PD—Phosphorus deficiency; PS—Phosphorus sufficiency.

The Scatter plot shows how different genotypes perform under *P*-sufficient (PS) and *P*-deficient (PD) conditions. Varieties in quadrant I (upper right) of the plot are considered as best genotypes under both, *P*-sufficient and *P*-deficient conditions. Genotypes in quadrants II and IV represent the best genotypes for PS and PD, respectively. While the genotypes in quadrant III are poor performers for both conditions (Fig. 1a,b).

For leaf dry weight (LDW), the genotypes JL 24, K 6, GG 20, ICGS 76, ICGV 00351, ICGV 87846 and ICGV 13121 are the best performers under both PS and PD, conditions. The genotype, ICGV 06146 under PD and ICGV 02266, ICGS 44 and ICGV 07240 under PS are identified as best performers for LDW. The genotypes ICGV 87846, ICGS 76, ICGV 13121, ICGV 00351, ICGV 05155 and ICGV 02266 are best performers under both PS and PD, whereas ICGS 44 and ICGV 13098 were best under PS and ICGV 07240, GG 20, ICGV 06146 were best under PD for the trait SDW. The outperformance for TT in both PD and PS was shown by ICGV 06040, ICGV 00350, ICGV 00351, ICGV 02266, ICGV 07240, ICGV 13115, ICGV 06146, ICGV 87846 and ICGS 76. Whereas, K 6 and GG 20 represented as best performers in PD and ICGS 44 and ICGV 13098 are best under PS for TT. By comparing the relative performance of genotypes for TE, genotype ICGV 06146 was best under PD and ICGV 87846 and ICGV 02266 were best under PS. But the genotypes ICGS 76, GG 20, K 6, ICGV 13121, ICGV 05155, ICGV 00351, JL 24 and ICGV 91114 are the best performers under both PS and PD for TE.

### Superior performing groundnut genotypes for P-efficiency related traits from pot experiment

Twenty groundnut genotypes are compared for four traits (LP%, LAP, RL and R:S ratio) under *P*-sufficient and *P*-deficient conditions and superior genotypes for each of these traits are identified based on the performance of genotypes under the two treatments (Table 4).

**Table 4:** Comparison of genotypes for P-efficiency traits, yield and P-indices.

Genotype	P- Indices			LAP		LP		RL		RS		PYP	
	APE	PE	PSF%	PS	PD	PS	PD	PS	PD	PS	PD	PS	PD
**Category 1**													
ICGV 00308	-0.02	1.20	−19.5	6.50	11.51	0.12	0.18	28.42	21.43	1.45	1.48	4.52	5.4
ICGV 02266	−0.02	1.27	−27.1	7.17	7.43	0.21	0.27	32.40	34.70	1.74	2.23	4.26	5.42
ICGV 05155	−0.01	1.09	−8.61	10.17	11.78	0.15	0.30	35.19	67.70	1.80	3.34	6.04	6.56
ICGV 06040	−0.01	1.07	−7.34	9.13	9.24	0.23	0.19	24.07	17.39	1.39	1.29	4.77	5.12
ICGV 06146	−0.01	1.06	−5.83	7.38	9.00	0.25	0.26	23.95	42.60	0.96	1.92	6.56	6.95
**Category 2**													
GG 20	0.01	0.83	16.86	8.41	13.36	0.16	0.23	25.78	28.80	1.21	2.02	3.08	2.56
ICGS 44	0.00	0.95	5.4	7.64	12.99	0.12	0.30	32.51	27.88	1.82	1.92	3.07	2.90
ICGV 13098	0.02	0.71	28.55	9.62	13.84	0.14	0.32	24.25	26.50	1.11	1.67	3.66	2.61
ICGV 13104	−0.01	1.09	−9.16	7.93	8.73	0.21	0.18	29.22	37.13	1.51	2.20	3.42	3.73
ICGV 13121	0.00	0.96	4.12	8.09	9.91	0.19	0.23	22.47	27.82	1.22	1.72	3.76	3.60
ICGV 91114	−0.01	1.24	−23.53	10.95	14.94	0.14	0.21	24.38	25.87	1.34	1.40	2.61	3.23
JL 24	0.03	0.69	30.83	12.54	13.80	0.16	0.16	29.92	31.37	1.90	2.37	5.39	3.73
**Category 3**													
ICGS 76	0.03	0.67	33.12	7.42	8.86	0.23	0.33	47.04	30.62	1.90	1.90	5.12	3.42
ICGV 00350	0.10	0.35	64.67	9.93	12.29	0.15	0.22	30.68	37.38	1.47	1.43	8.6	3.04
ICGV 00351	0.11	0.22	78.32	7.04	13.84	0.15	0.25	20.75	19.93	1.18	1.18	8.06	1.75
ICGV 07240	0.05	0.57	42.54	7.70	13.33	0.18	0.21	47.47	32.33	2.63	1.89	6.89	3.96
ICGV 13115	0.05	0.48	52.03	11.05	11.55	0.13	0.32	32.16	27.04	1.80	1.57	5.68	2.73
ICGV 87846	0.05	0.54	46.05	6.82	11.03	0.14	0.21	38.60	54.43	2.07	2.66	6.02	3.25
K 6	0.11	0.38	61.73	9.46	11.50	0.18	0.22	18.54	20.59	0.85	1.14	9.94	3.80
TAG 24	0.05	0.64	35.96	9.15	9.99	0.17	0.18	17.77	29.67	1.27	2.42	8.28	5.30

LP%—Leaf phosphorus (%), LAP—Leaf acid phosphatase (µM/hr/gm), RL—Root length (cm), R: S—Root: Shoot ratio, PYP—Pod yield per plant (g), APE—Agronomic phosphorus use efficiency; PE—Phosphorus efficiency; PSF (%)—Phosphorus stress factor percentage; PS- Phosphorus sufficient; PD- Phosphorus deficient.

The genotypes ICGV 06040 and 13104 accumulated higher *P* content in the leaves at 60 DAS under *P*-deficient condition (LP% of 0.23 and 0.21%, respectively) as compared to *P*-sufficient condition (LP% of 0.19 and 0.18%, respectively). For LAP, the genotypes ICGV 00351, 07240, 00308, 13098, 87846, ICGS 44 and GG 20 recorded higher phosphatase activity under *P*-deficient condition as compared to *P*-sufficient condition. For RL, the genotypes ICGV 05155, 06146, 87846, and TAG 24 recorded higher values under *P*-deficient conditions as compared to P-sufficient conditions. For the R:S ratio, the genotypes ICGV 05155, 02266, 06146, 13104, 87846, TAG 24 and GG 20 recorded higher readings under P-deficient condition as compared to *P*-sufficient condition.

### P-efficiency indices

The *P*-efficiency indices viz*.,* PSF (%), PE and APE are analyzed using mean pod yield data from *P*-sufficient and *P*-deficient conditions for both the years from the pot experiment. Significant differences are observed among the genotypes for *P*-efficiency indices. The PSF% varied from −56.75 to 78.32%, APE from −0.02 to 0.11, and PE from 0.22 to 1.27 (Table 4). Based on pod yield performance, the genotypes are classified into 3 categories- 1. Genotypes with high yield potential and high *P*-use efficiency (Efficient responding genotypes, ERG), 2. Genotypes with low yield potential and high *P*-use efficiency (non-efficient responding genotypes, NERG), and 3. Genotypes with high yield potential and low P-use efficiency (Efficient non-responding genotypes, ENRG).

Efficient responding genotypes (ERG) with high pod yield under both *P*-sufficient and *P*-deficient conditions include ICGVs 00308, 02266, 05155, 06040, and 06146. Non-efficient responding genotypes (NERG) with low pod yield under sufficient but less reduction/stable performance are GG 20, ICGS 44, ICGVs 13098, 13104, 87846 and K6. Efficient non-responding genotypes (ENRG) with high pod yield under sufficient but higher yield reduction under deficient conditions are ICGS 76, ICGVs 00350, 00351, 07240, 13115, 91114, 13121 and TAG 24. The comparison of PSF values when averaged for the three categories indicated a value of −13.68 for category 1 whereas it was 51.80 for category 3 indicating that efficient genotypes are least affected by P-deficient stress. Similarly, the PE and APE values are 1.14 and −0.01, respectively for ERG genotypes whereas, it is 0.48 and 0.07, respectively, for category ENRG.

### Stability analysis

**Figure 2:**
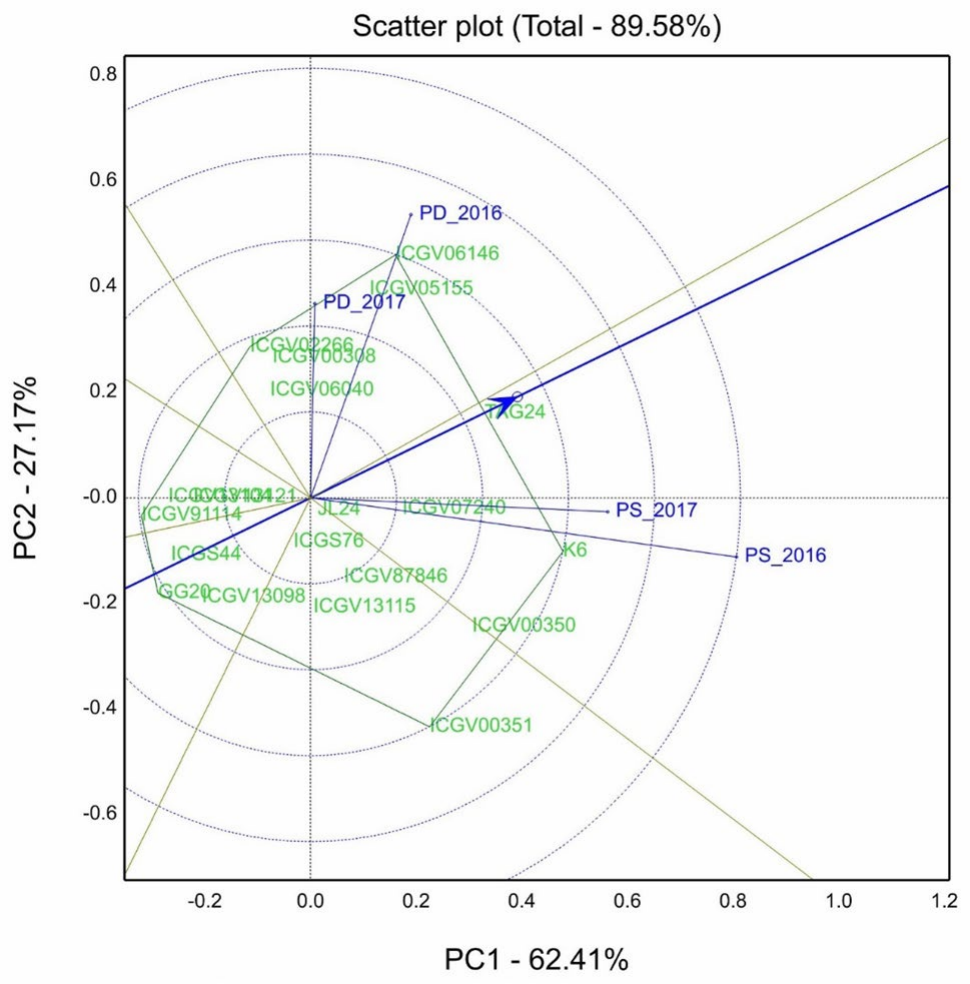
GGE biplot showing best genotypes for pod yield per plant under P-sufficient and P-deficient conditions under 2016 and 2017 pot experiment. PS_2016—P-sufficient condition under 2016; PS_2017- P-sufficient condition under 2017; PD_2016—P-deficient condition under 2016; PD_2017—P-deficient condition under 2017.

The stability analysis is conducted using the genotype and genotype × environment (GGE) interaction biplot technique proposed by^35^. The GGE biplot analysis was performed for pod yield per plant to identify the stable performers under *P*-sufficient and P-deficient conditions (Fig. 2).

In biplot analysis, a polygon is formed by fixing the vertex genotypes with straight lines and the rest of the genotypes positioned within the polygon. The splitting of GE interaction through GGE biplot analysis displayed that PC1 and PC2 of about 89.58% of GGE variation for pod yield per plant. The vertex genotypes are TAG 24, K 6, ICGV 00351, GG 20, ICGV 91114, ICGV 02266, and ICGV 06146 for pod yield per plant. The polygon view of biplot analysis showed that the genotypes are in seven sections and the test environments (*P*-sufficient and P-deficient) are in two sections. The four environments plotted formed two different mega-environments, one for *P*-sufficient condition and another for *P*-deficient condition.

An ideal genotype is close to the average environment coordinate (AEC), the small circle with an arrow, and has the least vector length. Genotype TAG 24 plotted closure to AEC with the least vector length from AEA indicates higher mean and stable pod yield per plant under *P*-sufficient and *P*-deficient conditions across both the years. Genotype K 6 and ICGV 00350 plotted near to *P*-sufficient environments and ICGV 06146 and ICGV 05155 plotted near to *P*-deficient environments with above-average pod yield performance and greater vector length from AEA indicates their superior performance under respective *P*-conditions.

### Correlation analysis

**Figure 3:**
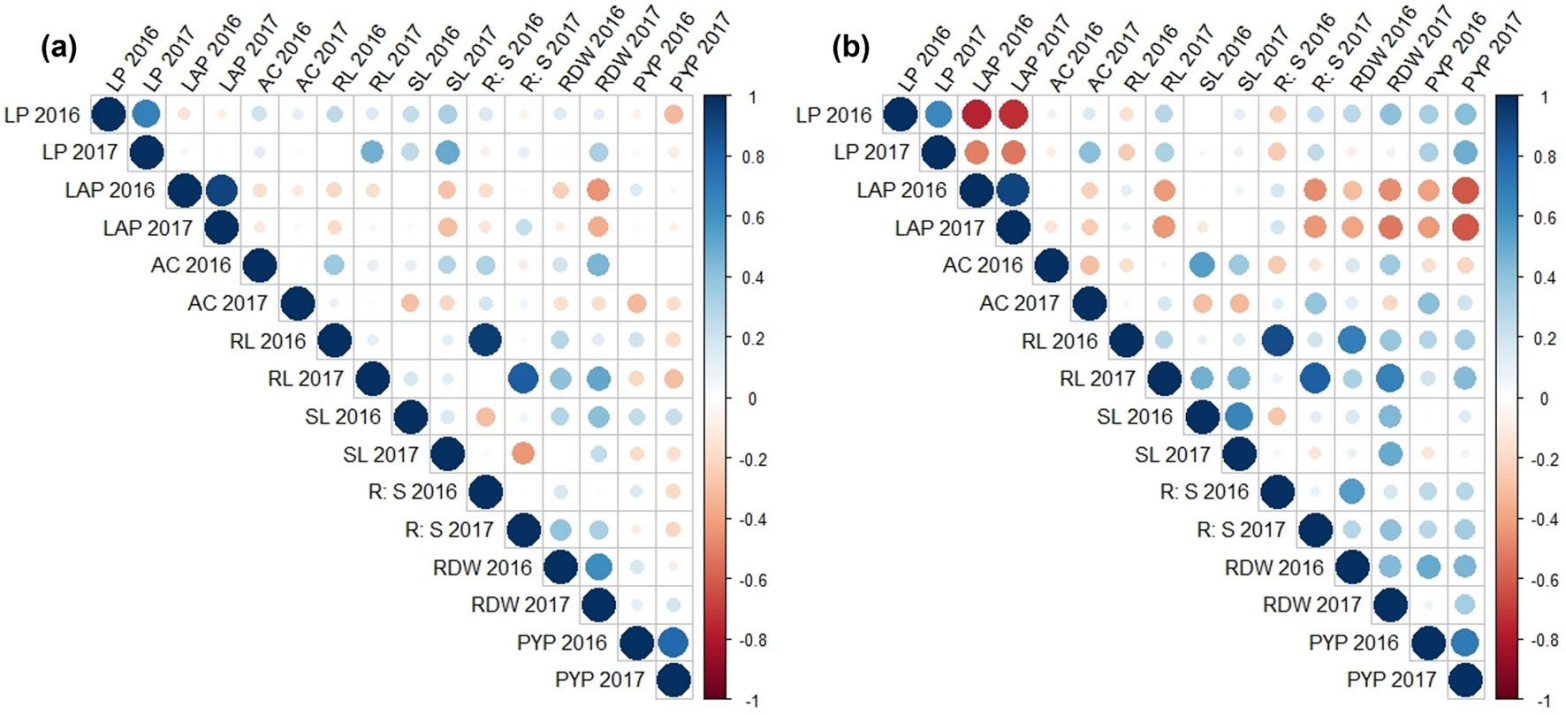
Correlation among the traits studied in pot (2016 and 2017) experiments under (**a**) P-sufficient condition (**b**) P-deficient condition. LP% 2016—Leaf phosphorus (%); LAP 2016– Leaf acid phosphatase (µM/hr/gm); AC 2016– Anthocyanin content (mg/g); RL 2016– Root length (cm); SL 2016 –S length (cm); R: S ratio 2016– Root: Shoot ratio; RDW 2016– Root dry weight (g); PYP 2016– Pod yield per plant (g); LP% 2017– Leaf phosphorus (%); LAP 2017– Leaf acid phosphatase (µM/hr/gm); AC 2017—Anthocyanin content (mg/g); RL 2017– Root length (cm); SL 2017–S length (cm); R: S ratio 2017—Root: Shoot ratio; RDW 2017– Root dry weight (g); PYP 2017—Pod yield per plant (g); Blue colour circles—size and thickness represent positive correlation; Red colour—size and thickness represents negative correlation.

The trait associations are studied using pot experiment data derived from P-sufficient and *P*–deficient conditions (Fig. 3a,b).

Under the *P*-sufficient condition, traits showed positive significant correlations. In 2016 pot experiment, R:S ratio 2016 showed significant association with RL 2016 (0.95***). In 2017 pot experiment, RL with LP% of 2017 (R^2^ = 0.47*); SL with LP% 2017 (R^2^ = 0.50*); R:S ratio with RL 2017 (R^2^ = 0.83***); RDW with RL 2017 (R^2^ = 0.52*) showed significant positive association. Whereas R:S 2017 showed significant negative association with SL 2017 (R^2^ = −0.44*).

Under P-deficient condition, in 2016 pot experiment, significant positive associations were observed between SL 2016 with AC 2016 (R^2^ = 0.55*); R:S ratio 2016 with RL 2016 (R^2^ = 0.88***) and RDW 2016 (R^2^ = 0.55*); RDW 2016 with RL 2016 (R^2^ = 0.68***) and PYP 2016 with RDW 2016 (R^2^ = 0.51*). In 2017 pot experiment, positive significant association was observed between SL 2017 with RL 2017 (0.45*); R:S ratio 2017 with RL 2017 (0.81***); PYP 2017 with RL 2017 (0.44*); RDW 2017 with RL 2017 (R^2^ = 0.67***) and SL 2017 (R^2^ = 0.50*) and PYP 2017 with LP 2017 (R^2^ = 0.48*). Whereas significant negative association was showed between LAP 2017 with LP 2017 (R^2^ = −0.52*); RDW 2017 with LAP 2017 (R^2^ = −0.52*); PYP 2017 with LAP 2017 (R^2^ = −0.61**).

### Phosphorus analysis in leaf and stem

The results revealed that there was a significant difference among the genotypes for total leaf and stem *P* content among the genotypes, however, no significant difference is observed for *P*-treatments (*P*-sufficient and *P*-deficient) and interaction effects (data not presented). Based on the total phosphorus in stem and leaf, two genotypes ICGV 00350 and 06146 are identified as the best and stable genotypes under both *P*-sufficient and *P*–deficient conditions.

## Discussion

The present study identified *P*-efficient groundnut genotypes, traits governing *P*-use efficiency, and their relationship with better water uptake by screening twenty groundnut genotypes in P-sufficient and P-deficient conditions. The combined ANOVA revealed significant genotypic differences for all the traits under study suggesting genetic variability for these traits in groundnut. G × E interaction effects were significant for all the traits under study except for AC indicating the differential response of genotypes over the environments. The G × Y, Y × T and G × Y × T interaction effects were non-significant for LP% and LAP indicating the absence of environmental influence on these traits and significant for all other traits indicating that these traits are highly influenced by the environmental conditions and to P treatments which indicate treatment is also an important component of the observed variation and it requires multi-season/location testing to make precise and robust selections. From the lysimeter experiment, there is a significant genotypic, treatment and genotype x treatment effect for all the traits under the study.

Surrogate traits that can be used as an indirect measure of *P*-efficiency in *P* limiting conditions to identify *P*-efficient genotypes are useful in a breeding program. Based on available literature four traits viz*.,* LP%, LAP, RL and R:S ratio were used to screen *P*-efficient genotypes^31,36–38^. The RL and R:S ratio are the two important factors that show more evident changes under *P* depriving conditions^36^^,^^37^^,^^38^. Under P-deficiency, there was an observed increase in root length to exploit phosphorus from deeper layers of soil. The phytohormones ethylene and auxin are involved in P- deficiency-induced root elongation^39^.

Under P-sufficient condition, vacuoles contain a storage pool of phosphorus whereas, *P* is concealed in chloroplast under deficient conditions, hence it expresses very low in leaf tissues^40^. The *P* accumulation in the leaves, root and shoot is a typical index for plant response under *P*-deficiency^7^^,^^41^. Another typical response of *P*-efficient genotypes to *P*-deficiency is increasing phosphatase enzyme to mineralize organic *P*^42^. Leaf acid phosphatase (LAP) can hydrolyze immobile into mobile orthophosphate anions under *P*- deficiency, LAP remobilizes *P* from metabolically fewer active sites such as old leaves and vacuoles to younger tissues^43^. The trait LAP activity is a good indicator for *P*-use efficiency in groundnut^31^ and it is also a good diagnostic tool for *P*-efficiency under P-deficiency conditions for many crops^44^. In the present study, the average LAP under the *P*-deficient condition (11.22 and 11.68 µM/hr/gm during rainy 2016 and 2017, respectively) was higher compared to the *P*-sufficient condition (8.48 and 8.93 µM/hr/gm during rainy 2016 and 2017, respectively). The increased activity of acid phosphatases in plant tissues under *P* deficient conditions were reported in different crops^30^^,^^26^^,^^45^^,^^46^^,^^47^^,^^48^^,^^27^ including groundnut^20^^,^^31^. Acid phosphatase is the obvious target for engineering of *P*-efficiency in many crops and overexpressing of this gene considerably enhances the *P* uptake efficiency in crop plants^49^. Transcriptome analysis of leaves and roots under *P*-sufficient and *P*-deficient conditions in soybean provided a significant role of the acid phosphatase gene in regulating P-use efficiency^50^.

The genotypes ICGV 00308, 02266, 05155, 06040 and 06146 also showed superior yielding ability as well as superior performance for one or more *P*-efficiency related traits (LP%, LAP, RL and R:S ratio) in separate pot experiments which further validates their ability to withstand *P*-stress. ICGV 00308 is a short-duration variety with good yield performance under normal culture conditions, while the rest are medium-duration varieties. The adaptation of superior genotypes under *P*-deficient conditions could be due to (i) better extraction of *P* in deficient soils; (ii) better source-sink mechanisms enabling them to transport *P* from leaves and stem to pods; (iii) tolerance of pods/kernels to lower levels of P^51^ and needs further study.

The *P* uptake efficiency is the ability to take more *P* from the soil under *P* limiting conditions and the *P* utilization efficiency is the ability to produce higher dry matter yield per unit of *P* absorbed from soil^52^. Three *P*-efficiency indices viz*.,* APE, PE and PSF were used in the present study to compare the twenty genotypes and categorize them into three separate categories- ERG, NERG and ENRG. The ERG category included ICGVs 00308, 02266, 05155, 06040, and 06146. Comparison of these ERGs with *P*-efficiency traits such as LP%, LAP, RL and R:S ratio indicated that the genotypes ICGVs 02266, 05155, 06146 were superior performing for multiple traits, ICGV 00308 is an efficient transporter due to high LAP activity and ICGV 06040 as accumulator due to its higher LP% under *P*-deficient condition in both years. In the present study, the pod yield per plant was recorded in pots grown under glasshouse condition/lysimeters. For better accuracy, there is a need to re-evaluate the identified best performing ERGs under field conditions where more precise information can be obtained especially for pod yield and associated traits as well as quality parameters.

From GGE biplot analysis, four environments plotted in separate sections forming two different mega-environments indicate the differential response of genotypes under *P*-sufficient and *P*-deficient conditions. The visualization of mega-environments in the biplot is useful in identifying specifically adapted genotypes under *P*-sufficient and *P*–deficient conditions. The polygon view of biplot identified vertex genotypes TAG 24, K 6, ICGV 00351, GG 20, ICGV 91114, ICGV 02266, and ICGV 06146 in the biplot for pod yield per plant indicates that these genotypes performed better in their respective environment. For selection, the stable P efficient genotypes are those which are close to AEC and occupy the least vector length from AEA. While selecting for adaptation, an ideal genotype should have both high mean performance and high stability within a mega environment^53^. From this study, genotype TAG 24 is close to AEC and occupied the least vector length from AEA. The genotypes K 6 and ICGV 00,350 and under P-deficiency genotypes and genotypes ICGV 06146 and ICGV 05155 with above-average pod yield performance and greater vector length from AEA indicate their superior performance under respective *P*-conditions. The best stable genotype identified in this study can be used to improve phosphorus uptake by combining multiple trait performance with more adaptability.

For different physiological traits studied in the lysimeter, the performance of genotypes under *P*-sufficient and *P*-deficient conditions were presented in a scatter plot. The genotypes GG 20, ICGS 76, ICGV 87846, ICGV 00351, K6, ICGV 13121 and JL 24 performed well for LDW; ICGV 06040, ICGV 05155, ICGS 76, ICGV 87846, ICGV 00351, ICGV 13121 and ICGV 02266 performed well for SDW; ICGV 00350, ICGV 07240, ICGV 13115, ICGV 06146, ICGS 76, ICGV 87846, ICGV 00351, ICGV 02266 performed well for TT and genotypes ICGV 05155, GG 20, ICGS 76, ICGV 00351, K 6, ICGV 13121, JL 24, ICGV 91114 performed well for TE under both *P*-sufficient and *P*-deficient conditions as they occupied quadrant I.

All genotypes showed higher value for LDW under P-sufficient condition compared to *P*-deficient condition except two lines ICGV 06146 and TAG 24. Similarly, expect two genotypes GG 20 and ICGV 06146, remaining genotypes showed an increased trend of SDW in *P*-sufficient condition compared to deficient condition. The TT and TE were higher in the *P*-sufficient condition compared to the *P*-deficient condition expect four genotypes for TT and two genotypes for TE. *P* is required for the opening mechanism of stomata^54^^,^^55^ and its deficiency reduces stomatal aperture. Another effect of *P*-deficiency is the lowering of stomatal density in *P*-deficient leaves resulting in higher stomatal resistance^56^. Atkinson and Davison (1972)^57^ observed that the leaves of *Arctium minus* Bernh. plants grown under *P*-deficient conditions had smaller cells and a much dense layer of hair on the leaf surface as compared to controls. These mechanisms of stomatal resistance may be the reason for the reduction in total transpiration and transpiration efficiency in *P*-deficient soils.

Correlation analysis under *P*-sufficient and *P*-deficient conditions revealed significant associations for some traits. A significant negative association was observed among LAP and LP% under *P*-deficient conditions for both 2016 and 2017. The negative association between LAP and LP% has earlier been reported in groundnut^31^ and soybean^58^. The LP% gets significantly decreased under *P*-deficient conditions leading to an increase in LAP content^59^^,^^60^. This is due to developing tissues of the younger plant and *P*-stressed plants usually show higher acid phosphatase activity, which requires *P* supply from older tissues by hydrolyzing *P* from organic forms to inorganic and remobilizing it to the apical growing parts^61^^,^^62^^,^^58^.

Root traits are important for the improvement of *P*-use efficiency as they directly affect *P* absorption^63^. RL showed a positive significant correlation with the R:S ratio for both the years 2016 and 2017 and with root dry weight only in 2017 under *P*-sufficient and *P*-deficient conditions. The limited supply of *P* leads to increased R:S ratio, root length, root volume and root hair density^64^^,^^65^^,^^66^^,^^67^.

The results of the total leaf and stem *P* content of seven selected genotypes revealed significant differences among genotypes. However, the genotype × treatment interaction effects were non-significant indicating that the genotypes performed consistently under *P*-sufficient and *P*-deficient conditions. The study identified genotypes ICGV 00350 and ICGV 06146 as consistent performers for total leaf and stem P content.

## Materials and methods

### Greenhouse experiment

Twenty groundnut genotypes (17 Spanish Bunch and 3 Virginia Bunch types) collected from International Crops Research Institute for the Semi-Arid Tropics (ICRISAT) were used in the study (Table 5). The genotypes are named as ICGV which stands for ICRISAT Groundnut Variety followed by number. Both field and pot experiments were carried out in accordance with the institute guidelines and necessary permission was obtained to collect the groundnut kernels. The experiment was conducted in pots using factorial randomized complete block design (F-RCBD) with two replications and two treatments of *P* (*P*-sufficient and *P*-deficient) at Regional Agricultural Research Station, Tirupati, Andhra Pradesh, India during rainy 2016 and 2017. Soil testing showed that *P*-sufficient and *P*-deficient soils had 78.6 kg/ha and 23.5 kg/ha of soil available P_2_O_5_, respectively. According to USDA (2001) report on soil phosphorus, the available P in the range of 5–10 ppm in soil indicates *P* deficiency and > 20 ppm indicates rich in *P*. In this study, the available *P* in *P*-sufficiency soil is 20 ppm and *P*- deficient soil has 6 ppm values.

Plastic pots of 5 kg capacity were filled with soil mixture and six kernels per pot were planted. Thinning was done after ten days of sowing and four plants per pot were maintained across replications and *P*-treatments. Leaf phosphorus (LP%), leaf acid phosphatase (LAP) (µM/hr/gm), anthocyanin content (AC) (mg/g), root length (RL) (cm), shoot length (SL) (cm), root dry weight (RDW) (g), root: shoot ratio (R:S ratio) and pod yield per plant (PYP) (g) are measured in both *P* conditions separately.

**Table 5:** Details of groundnut genotypes used in this study.

S. No	Genotype	Cross/Pedigree	Key features
	**Growth Habit: Spanish Bunch type genotypes**		
1	ICGV 91114	(ICGV 86055 × ICGV 86533)	Susceptible to rust and late leaf spot (LLS) and drought tolerant
2	ICGV 00308	(ICGV 95244 × ICGV 96223)	Drought tolerant
3	JL 24	(Selection from 'EC-94943')	Wider adaptability and susceptibility to LLS and Rust
4	Kadiri 6	(JL-24 × Ah-316/S)	Susceptible to LLS and rust
5	TAG 24	(Selection from TGS-2 (TG-18A × M 13) × TGE-1 (Tall mutant × TG-9))	Semi-dwarf plant type and high harvest index (50–55%)
6	ICGS 44	(Robut-33–1-1–5-B1-B1-B1-B1 (Selection from natural hybrid population of Robut 33–1))	Tolerant to early leaf spot (ELS), peanut bud necrosis disease(PBND) and mid-season drought
7	ICGV 00,50	(ICGV-87290 × ICGV-87846)	Tolerant to LLS and rust, drought tolerant
8	ICGV 00,351	(ICGV 87290 × ICGV 87846)	Tolerant to LLS and rust, drought tolerant
9	ICGV 02266	((ICGV 88414 × USA 63) CF5-68 × ICG 6330)	Higher pod and fodder yield and suitable for *Rabi and* Summer cultivation, drought tolerant
10	ICGV 07240	((ICGV 92069 × ICGV 93184) SIL 4 × ICGV 98300)	Moderate drought tolerant and less leaf minor incidence
11	ICGV 05155	(ICGV 99160 × ICGV 99240)	High oil content, tolerant to rust and LLS
12	ICGV 06146	((ICGV 92069 × ICGV 93184) × (ICGV 96246 × 92 R/75))	High oil content, tolerant to rust and LLS
13	ICGV 13104	(ICGV 00350 × ICGV 06420)	High oil content
14	ICGV 13115	(ICGV 00350 × ICGV 06420)	High oil content
15	ICGV 13098	(ICGV 00350 × ICGV 06,420)	High oil content
16	ICGV 13121	((ICGV 01031 × ICG 14985) × ICGV 04044)	Tolerant to preharvest Aspergillus infection
17	ICGV 06040	((ICGV 92069 × ICGV 93184) × (NC Ac 343 × ICGV 86187)S23)	High Fe & Zn concentration in the kernels, and heat stress tolerance
	**Growth Habit: Virginia Bunch type genotypes**		
	Genotype	Pedigree	Remarks
1	ICGV 87846	(CS 9 × ICGS 5)	High yielding, drought tolerance
2	ICGS76	(TMV 10 × Chico)	Medium duration adapted to low input rainfed condition. Tolerant to mid-season drought and bud necrosis
3	GG 20	(GAUG-10 × R-33–1)	High yielding, medium bold kernels

ICGV = ICRISAT Groundnut Variety; ICGS = ICRISAT Groundnut Selection; LLS = Late leaf spot.

At the final harvest, root samples from each pot were washed under running tap water to remove soil, and root length is measured with a centimeter scale. Root samples are oven-dried at 80 °C for 48 h to record root dry weight. The shoot length in cm is measured with scale for each plant at the final harvest. At harvest, pods are separated from the plants, air-dried in the oven to about 8% moisture, and pod dry weight was recorded in grams.

### Estimation of LAP and LP%

At 60 days after sowing (DAS), the LAP enzyme activity has been assessed using the para-nitrophenyl phosphate (p-NPP) method^30^. The leaf samples for analysis were collected from index leaves *i.e.*3rd and 4th mature leaves from the apical bud^68^. Fragments of freshly collected leaves (100 mg) were washed with water and incubated for 20 min in a water bath at 30 °C by adding 8 ml of 0.25 mmol/L p-NPP in 0.1 ml (pH 4.0) sodium acetate buffer. The intensity of the yellow color is read against a blank (without leaf sample) at 405 nm using Ultraviolet–Visible (UV-Vs) spectrophotometer (GENESYS 10S UV–Vis, USA). The p-NPP quantity is estimated from the percent absorbance and the enzyme activity is expressed as μmol of p-NPP per hour per g (µM/hr/gm) of fresh tissue. After acid phosphatase estimation, the remaining index leaves are dried in a hot air oven at 65 °C for 72 h followed by weighing and grinding the sample. The ground samples are analyzed for LP% as per the procedure of^69^.

### Estimation of anthocyanin content

Anthocyanin content of leaves is estimated by the method proposed by^70^. In a pestle and mortar, 500 mg of the third quadrifoliate leaf tissues were grounded with 10 ml of 1% methanol in three biological replications. After centrifugation of the homogenate mixture, the resultant supernatant is diluted with 1% HCl-methanol to 50 ml. The intensity of absorption of diluents is measured at 530 nm using a spectrophotometer and anthocyanin contents are expressed in mg per gram (mg/g) of fresh weight.

### Lysimeter experiment

The same set of twenty genotypes was tested under the lysimetric setup (http://gems.icrisat.org/phenotyping/) at International Crops Research Institute for the Semi-Arid Tropics (ICRISAT), Patancheru, India (17°30′N; 78°16′E; altitude 549 m) during post-rainy 2017–18 (Table 5). Lysimeter set up is equipped with PVC cylinders of 1.2 m deep and 20 cm diameter placed in below ground trenches to facilitate individual soil profiles for each place under study. It has a rain-out shelter to protect the plants from rainfall. The lysimeter tubes are filled with readily available loamy soil that is low in P content (2.5 ppm) from the ICRISAT field. The protocol for lysimeter soil preparation & filling, spacing arrangement, growing and weighing plants were followed according to^71^and^72^.The experiment was planned in a complete randomized block design. One block was assigned to a phosphorus sufficient (PS) and another block for phosphorus-deficient (PD). After the final thinning, P-sufficient treatment received DAP (5 g/cylinder) and potash (2 g/cylinder) and *P*-deficient treatment involved the application of urea (2 g/cylinder) and potash (2 g/cylinder). The lysimetric cylinders are separated from one another by approximately 2 cm so that the planting density of groundnut is about 21 plants per square meter, a density very similar to the field planting (20–25 plants/m^2^). During the experiment, the data logger was positioned within the plant canopy to record the day/night temperatures and relative humidity, which fluctuated under the natural day-night oscillations with an average of 31.7/15.5 °C and 40/85% respectively.

Thinning was done at 4 weeks after sowing to maintain 2 plants per cylinder. Each cylinder received 500 ml of water twice a day until 14 DAP (days after planting) and 500 ml on alternate days thereafter until 28 DAS. The cylinder surface is covered with a 2-cm layer of plastic beads to avoid soil evaporation^73^. A day before weighing, the cylinders were irrigated (2 L per cylinder) and allowed to drain the excess water overnight to reach field capacity as described in^73^. The next morning, cylinders were weighed by lifting them with a block chained pulley using an S-type load cell (Mettler-Toledo, CSE 100, Geneva, Switzerland) and this weighing process was done every week until crop maturity. Based on the differences in cylinder weights, transpiration of the plants was assessed in each cylinder. From the week the weighing began until the harvest, the plants of P-sufficient and deficient treatments were maintained at 80% of field capacity by weekly re-watering. At the end of the experiment, plants were harvested, the crop residuals dried at 60 °C in an oven for 72 h and the above-ground biomass and vegetative dry biomass were weighed (KERN 3600 g; 0.01 g precision balance, Kern & Sohn GmbH, Balingen, Germany). At the final harvest, leaf and stem samples are oven-dried at 80 °C for 48 h and weighed separately as leaf dry weight (LDW) and stem dry weight (SDW). The total transpiration is measured as the sum of each week's transpiration for a particular cylinder considering the amount of water compensated each week against transpiration.

The total transpiration (TT) and transpiration efficiency (TE) was measured using the formulas

$${\text{TT }}\left( {\text{g}} \right) \, = \left[ {\left( {{\text{W1 }}\left( {\text{g}} \right) \, {-}{\text{ W2 }}\left( {\text{g}} \right)} \right) \, + {\text{ water added}}} \right]/{\text{n}}$$

$${\text{TE }}\left( {{\text{g}}/{\text{kg}}} \right) \, = {\text{ shoot biomass}}/{\text{water transpired}}$$

Where W1- Initial weight of the cylinder; W2- Final weight of the cylinder, n- Number of plants/cylinders.

### P-efficiency indices

P-efficiency indices such as, phosphorus stress factor (PSF), *P*-efficiency (PE) and agronomic *P*-use efficiency (APE) of genotypes were calculated as per the formula given below^20^.

$$1. \; \mathrm{ Phosphorus\, stress \,factor \%}=100\mathrm{ X }\frac{({\mathrm{PY}}_{\mathrm{Ade}}-{\mathrm{PY}}_{\mathrm{Def}})}{{\mathrm{PY}}_{\mathrm{Ade}}}$$

$$2. \; \mathrm{ P\, efficiency}= \frac{{\mathrm{PY}}_{\mathrm{Def}}}{{\mathrm{PY}}_{\mathrm{Ade}}}$$

$$3. \; \mathrm{ Agronomic\, P\, use\, efficiency}= \frac{\left({\mathrm{PY}}_{\mathrm{Ade}}-{\mathrm{PY}}_{\mathrm{Def}}\right)}{{\mathrm{AP}}_{\mathrm{App}}}$$

PY_Ade_–pod yield under phosphorus-sufficient condition, PY_Def_–pod yield under phosphorus-deficient condition, AP_App_–a difference in the amount of *P* applied between treatments (sufficient and deficient conditions).

### Phosphorus analysis in leaf and stem

Based on the 2016 and 2017 pot experiments, genotypes are categorized into poor performers (ICGV 00350 and ICGV 00351), intermediate performers (GG 20, TAG 24 & ICGV 13104) and good performers (ICGV 02266 and ICGV 06146) and these seven genotypes are used for estimation of total *P* in leaf and stem. The leaf and stem samples of these genotypes collected in three replications from *P*-sufficient and *P*-deficient treatments from the lysimeter experiment are analyzed for leaf and stem total *P* content by following the sulfuric acid-selenium digestion method^74^.

### Statistical analysis

The data recorded from both the experiments (pot and lysimeter) are subjected to two-way analysis of variance (ANOVA) using residual maximum likelihood (REML) method using SAS version 9.4 (SAS Institute Inc., Cary, NC, USA) (https://www.sas.com/en_us/home.html). Best Linear Unbiased Predictors (BLUPs) were assessed for genotypes (G), P-treatments, between years and their interactions from combined analysis of variance and calculated pairwise comparisons using t-statistic (LSD). Pearson correlation coefficients were illustrated using the “Corrplot” package in R V 3.0 software. GGE biplot analysis and scatter plot are illustrated by^75^ using GENSTAT version 15.0 (VSN International Ltd. Hemel Hempstead, UK) (https://vsni.co.uk/software/genstat).

## Conclusion

Adaptation of groundnut genotypes for *P*-deficient conditions helps to sustain the production as well as to reduce the burden on depleting reserves of rock phosphate. The present study identified P-efficient genotypes viz., ICGVs 06146, 07240, 02266, 06040, 87846, 05155, 00308, GG 20 and TAG 24 based on comparison under *P*-sufficient and deficient conditions for *P*-uptake surrogate traits, LP%, LAP, RL and R:S ratio. Based on *P*-efficiency indices, the genotypes ICGV 00308, 02266, 05155, 06040 and 06146 were categorized as ERG (effecient responding genotypes) genotypes for their lower *P*-stress factor (PSF) and agronomic phosphorus use efficiency (APE) values along with *P*-efficiency values of  ≥ 1. From lysimeter data, ICGV 06146 showed better performance for LDW, SDW and TT. So, ICGV 06146 appeared as the most promising for *P*-deficient soils in terms of *P*-related, yield, agronomic and physiological traits. The identified genotypes needs to be evaluated further under field conditions of low and high *P* to corroborate the pot results with the field. Furthermore, research efforts are required to assess the genetic variability for *P*-use efficiency traits in a large germplasm set, identify the quantitative trait loci and candidate genes responsible for *P*-use efficiency under *P*-deficient soils. The selected *P*-efficient genotypes can be used to identify the *P*-efficiency regulating genes. In the future, improved varieties of groundnut can be developed through breeding programs using *P*-efficient genotypes.

## Data Availability

All data generated or analyzed during this study are included in this published article in the tables and figures and no additional data is available.
