# Author Correction: Genetic approaches for assessment of phosphorus use efficiency in groundnut (*Arachis hypogaea* L.)

**DOI:** 10.1038/s41598-023-28050-z

**Published:** 2023-01-19

**Authors:** Sai Rekha Kadirimangalam, Yashoda Jadhav, K. V. Nagamadhuri, Latha Putta, Tharanya Murugesan, Murali T. Variath, Anil Kumar Vemula, Surendra Singh Manohar, Sunil Chaudhari, Sunita Choudhary, Jana Kholova, Janila Pasupuleti

**Affiliations:** 1 International Crops Research Institute for the Semi-Arid Tropics (ICRISAT), Hyderabad, Telangana 502324 India; 2 Institute of Frontier Technology, Regional Agricultural Research Station, Acharya N.G. Ranga Agricultural University, Tirupati, Andhra Pradesh 517502 India

Correction to: *Scientific Reports* 10.1038/s41598-022-24016-9, published online 13 December 2022

The original version of this Article contained a typo in the Abstract.

“Production of phosphorus efficient genotypes in groundnut can improve and also reduces environmental pollution.”

now reads:

“Production of phosphorus efficient genotypes can reduce environmental pollution”.

The original Article has been corrected.

